# Silk Fibroin Microneedle Patches for the Treatment of Insomnia

**DOI:** 10.3390/pharmaceutics13122198

**Published:** 2021-12-20

**Authors:** Zhenzhen Qi, Jiaxin Cao, Xiaosheng Tao, Xinyi Wu, Subhas C. Kundu, Shenzhou Lu

**Affiliations:** 1 National Engineering Laboratory for Modern Silk, College of Textile and Clothing Engineering, Soochow University, Suzhou 215123, China; 20204215016@stu.suda.edu.cn (Z.Q.); 20185215038@stu.suda.edu.cn (J.C.); 20194215005@stu.suda.edu.cn (X.T.); 2015408103@stu.suda.edu.cn (X.W.); 2 3Bs Research Group, I3Bs Research Institute on Biomaterials, Biodegrabilities and Biomimetics, University of Minho, 4710-057 Braga, Portugal; kundu@i3bs.uminho.pt; 3 Headquarters of the European Institute of Excellence on Tissue Engineering and Regenerative Medicine, University of Minho, AvePark, Barco, 4805-017 Guimaraes, Portugal

**Keywords:** silk fibroin, microneedles, melatonin, transdermal sustained release, insomnia

## Abstract

As a patient-friendly technology, drug-loaded microneedles can deliver drugs through the skin into the body. This system has broad application prospects and is receiving wide attention. Based on the knowledge acquired in this work, we successfully developed a melatonin-loaded microneedle prepared from proline/melatonin/silk fibroin. The engineered microneedles’ morphological, physical, and chemical properties were characterized to investigate their structural transformation mechanism and transdermal drug-delivery capabilities. The results indicated that the crystal structure of silk fibroin in drug-loaded microneedles was mainly Silk I crystal structure, with a low dissolution rate and suitable swelling property. Melatonin-loaded microneedles showed high mechanical properties, and the breaking strength of a single needle was 1.2 N, which could easily be penetrated the skin. The drug release results in vitro revealed that the effective drug concentration was obtained quickly during the early delivery. The successful drug concentration was maintained through continuous release at the later stage. For in vivo experimentation, the Sprague Dawley (SD) rat model of insomnia was constructed. The outcome exhibited that the melatonin-loaded microneedle released the drug into the body through the skin and maintained a high blood concentration (over 5 ng/mL) for 4–6 h. The maximum blood concentration was above 10 ng/mL, and the peak time was 0.31 h. This system indicates that it achieved the purpose of mimicking physiological release and treating insomnia.

## 1. Introduction

Insomnia is a common physiological and psychological disease characterized by difficulty falling asleep or maintaining normal sleep [1]. Long-term insomnia has severe adverse effects on people’s everyday life and work and can even cause serious accidents [2]. In the pharmacological treatment of sleep disorders, drugs with a short half-life are to be used to reduce the hangover effect the next day [3]. At the same time, drug dependence and tolerance caused by long-term medication should be avoided. Melatonin (MT) was identified initially in bovine pineal glands in 1958 [4,5]. Early clinical experiments [6] proved that melatonin had a sleep-promoting role and was primarily secreted according to a circadian rhythm. The physiological role of melatonin in regulating circadian rhythm is mediated by a high-affinity G protein-coupled receptor [7]. As the aging process occurs, the secretion of endogenous melatonin is less and less, leading to a gradual imbalance of biological rhythms and indirectly increasing the incidence of some other senile diseases. Compared with other drugs, exogenous melatonin has no dependence or adverse effects such as hangover effect on the following day [8], with a short half-life [9]. Side effects on humans after low-dose melatonin administration are low [10]. Recently, researchers demonstrated that exogenous melatonin could improve sleep quality [11,12]. Therefore, melatonin is an ideal drug for the treatment of insomnia.

Exogenous melatonin is usually administered orally. Due to the short half-life of melatonin, the drug effect decreases rapidly after entering the body. It is difficult to maintain an adequate drug concentration in the blood for a long time [13]. Oral administration has a liver first-pass effect, leading to low bioavailability of oral melatonin [14,15]. Researchers are working on oral sustained-release melatonin tablets, using some excipient agents such as hydroxypropyl methylcellulose, polyvinylpyrrolidone, and sodium alginate to prolong the time of drug entry into the human body [16,17]. However, the liver first-pass effect still exists. Clinically, intranasal administration of melatonin wrapped in nanoscale liposomes have been studied in Wistar rats. Compared with intravenous administration of the same amount of melatonin, melatonin encased in liposomes can induce sleep and delay systemic circulation [18]. However, this prolonged-release melatonin formulation is difficult to use, and the dosage is difficult to control. Transdermal delivery of melatonin may be superior to oral delivery of melatonin in improving the maintenance of disturbed sleep cycles [19]. A transdermal patch delivered melatonin in eight healthy subjects [19]. Compared with oral melatonin, the patch steadily increased plasma melatonin concentration, but the drug release rate was slower, reaching the highest plasma concentration 8.5 h later. Another group of researchers investigated the pharmacokinetics of solid, melatonin-carrying lipid nanoparticles by oral or transdermal pathway [20]. Compared with the oral route, the transdermal way of melatonin had higher bioavailability and blood level (50 pg/mL) maintenance time. However, it took several hours for the patch to release melatonin at detectable plasma levels, which did not ensure fast sleep for insomnia patients.

Microneedles (MNs) are thought to be a cross between a transdermal patch and a hypodermic needle, capable of penetrating the outermost layer of the skin (the cuticle) for local delivery. This allows drug molecules to enter the superficial dermis and be transported through capillaries throughout the body to achieve a similar effect to subcutaneous injection [21,22,23]. Because of its advantages of low cost, convenient use, painless use and high efficiency, it has shown great potential in the field of transdermal drug delivery. The administration of MNs varies according to the types of MNs. Dissolved MNs directly release the drug into the skin simultaneously, while swelling MNs absorb the skin’s interstitial fluid and adjust the time and rate of administration by adjusting the swelling properties to achieve sustained release [24]. MNs have successfully delivered various drugs, such as metronidazole, caffeine, insulin, and others [25,26]. Silk fibroin (SF), as a natural protein, comes from a wide range of sources and has excellent mechanical properties and biological safety [27,28,29], showing great potential in drug delivery applications [30]. Tsioris et al. [31] made MNs using silk fibroin as raw material for the first time and proved that silk fibroin microneedles could successfully penetrate the skin and be used as a carrier for drug delivery and storage. Tao et al. [32] successfully prepared a silk fibroin microneedle patch that directly released the drug to the tumor site, which could significantly reduce the tumor size and improve the survival rate of the mice. Our previous work showed that silk fibroin microneedles with different swelling degrees could be prepared by mixing various minor molecule additives with silk fibroin [33,34,35]. The drug release mode can be controlled, and the drug release rate can be regulated based on the MN platform. Insulin can be successfully loaded into microneedles to obtain insulin microneedles loaded with silk fibroin. In vitro and in vivo verifications exhibited as compared to conventional injection, silk fibroin microneedles can maintain insulin bioactivity and successfully achieve the hypoglycemic effect of diabetic rats [34,35].

To improve the bioavailability of exogenous melatonin and accelerate the rate of melatonin entering the blood, we used silk fibroin to prepare microneedles loaded with melatonin. In this paper, the swelling degree of the MN was adjusted by changing the amount of proline to obtain the swelling microneedles with rapid release in the early stage and stable release for 4–6 h in the later stage. The drug-release rates of melatonin-loaded MNs were measured in vitro and in vivo. The efficacy of melatonin-loaded microneedles was evaluated on the constructed SD rats with insomnia conditions. These set-up rats were effectively treated for maintaining a relatively long and stable sleep state.

## 2. Materials and Methods

### 2.1. Experimental Materials

Fresh silkworm cocoon shells Suzhou Xiancan silk Biotechnology Co., Ltd. (Suzhou, China); LiBr: Tiancheng Chemical Co., Ltd. (Yanzhou, China); Na_2_CO_3_, Na_2_HCO_3_, KCl, NaCl: Sinopharm Chemical Reagent Co., Ltd. (Shanghai, China); Methanol: Jiangsu Qiangsheng Functional Chemistry Co., Ltd. (Suzhou, China); Isopropyl alcohol, melatonin (MT): Shanghai Alading Biotechnology Co., Ltd. (Shanghai, China); p-Chlorophenylalanine: Ark Pharm (Chicago, IL, USA); SD male rats: Animal Experiment Center of Soochow University were obtained for our experiments.

### 2.2. Preparation of Silk Fibroin Solution

The 80 g cocoons were placed in 4000 mL of 0.3 wt.% Na_2_CO_3_, 0.1 wt.% NaHCO_3_ solution and boiled at 100 °C. The process was repeated three times, and the cocoons were scrubbed to remove the sericin completely. The silk fibroin fiber was dried in an oven at 60 °C. Fifteen-gram degummed silk fibroin fibers were weighed and dissolved in lithium bromide solution (9.3 M) at 65 °C for 1 h. After cooling to room temperature, the solution was placed in a dialysis bag and placed in deionized water at 4 °C for 3 to 4 days. The obtained silk fibroin (SF) solution was placed in a refrigerator at 4 °C for further use for a shorter duration.

### 2.3. Preparation of MNs

**Figure 1:**
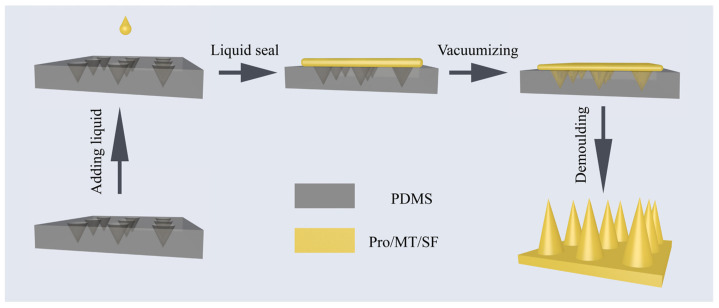
The preparation process of melatonin-loaded silk fibroin microneedle (MT/SF@MNs): the mixture was poured into a polydimethylsiloxane microneedle mold, evacuated 3 times to remove the bubbles, and then dried in a constant temperature and humidity room for 8 h (25 °C, 55%RH) and demolded to obtain MNs.

Five milligrams of melatonin was weighed, and 10% isopropyl alcohol was added to form a 0.5 mg/mL melatonin solution. The mixture of proline/melatonin/silk fibroin (50 mg/mL) was mixed with a mass ratio of 0/1/1000, 10/1/100, 20/1/100, 30/1/100, 50/1/100 (*w/w*). The preparation process of MNs is shown in Figure 1. The mixture was poured into a polydimethylsiloxane microneedle mold, evacuated 3 times to remove the bubbles from the solution on the mold, and then dried in a constant temperature and humidity room for 8 h (25 °C, 55% RH) and demolded to obtain MNs (MT/SF@MNs replaced below). The prepared pure silk fibroin MNs without proline were sprayed with 90% methanol on the surface and used as the control group of MNs with the crystal structure of Silk II [36].

### 2.4. X-ray Diffraction and Fourier Transform Infrared Spectroscopy

The samples to be tested were cut into a fine powder, and the powder was collected after sieving through an 80-mesh sieve. The crystal structure was tested by X ‘Pert PRO MPD diffractometer, and the material’s Wide Angle X-ray diffraction curve was obtained. The instrument parameters were set as follows: 10°/min scanning speed, 40 kV stable unfluctuating tube voltage, 30 mA steady unfluctuating tube current, and the diffraction intensity curve recorded between a 5° and 45° diffraction angle.

The powder to be tested was mixed with potassium bromide, and the tablet pressing mechanism was used to prepare the tablet following the requirements. The absorbance in the range of 400~4000 cm^−1^ was measured by Nicolet 5700 INFRARED spectroscopy analyzer, and the infrared absorption spectra were obtained.

### 2.5. Mechanical Properties and Penetration Properties

**Figure 2:**
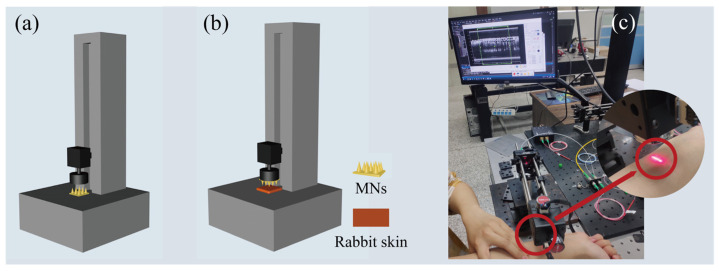
The schematic diagram for processing of mechanical test of melatonin-loaded silk fibroin microneedle (MNs): (**a**) schematic diagram of compression performance test, (**b**) schematic diagram of penetration performance test, and (**c**) schematic diagram of mechanical test process of MN penetration into human skin.

The MNs were cut into a 3 × 3 array (N = 5), and the tip of the MNs was placed upward on the TMS-PRO texture instrument to test the compressive breaking strength of a piece of MNs (Figure 2a) and then divided by 9 to obtain the compressive breaking strength of a single needle. The initial force was 0.05 N, the compression ratio was 80%, and the test rate was 10 mm/min.

The backing layer of the 15 × 15 MN array (N = 5) was pasted on the probe board of the force-sensing component of the texture analyzer. Rabbit skin was placed on the operating table below and dropped at a 10 mm/min rate to test the rabbit skin penetration force of the single MN (Figure 2b).

To evaluate the penetration performance of the MN patch, we applied the patch (15 × 15) to the hand of a 25-year-old human volunteer (co-author, Jiaxin Cao). A scanning frequency source optical coherence tomography (OCT) imaging system (prototype device of Suzhou Institute of Biomedical Engineering and Technology, Chinese Academy of Sciences) was used to obtain the depth information of human tissues and observe the state of microneedle insertion into the skin by using the low-coherence principle of light (Figure 2c). Among them, the scanning width was 5 mm, the depth was 6 mm, the central wavelength was 1310 nm, and the imaging frame rate was 166 Hz.

### 2.6. Dissolving and Swelling Capacities

Prepared MNs weighing 0.1 g were weighed (5 parallel samples in each group) and placed in phosphate-buffered solution (PBS, pH = 7.4) at a bath ratio of 1:100 (*w*/*v*) for 24 h at 37 °C. After soaking, the surface moisture of the MN was removed, and its apparent mass was weighed and denoted as m_1_. The remaining solution was centrifuged at 3500 rpm for 15 min, and the supernatant was taken for spectrophotometer reading. The absorbance A was measured at 278 nm by ultraviolet spectrophotometer, and the concentration C was obtained by conversion according to the absorbance–concentration standard curve drawn by the previous measurement. According to Formulas (1) and (2), the swelling rate of MNs and the loss rate of silk fibroin were calculated, respectively:

(1) $$\text{Swelling ratio}\;(\%)=\frac{m_{2}-m_{1}}{m_{1}}\;\times \;100\%$$

(2) $$\text{Dissolving ratio}\;(\%)=\frac{C\;\times \;V}{m_{1}\;\times \;N\;\times \;[\frac{1}{1+S}]}\;\times \;100\%$$

where m_1_ is the initial mass of the MN (g); m_2_ is the mass of MN after swelling (g); N is the solid content of MNs (the ratio of constant weight to initial mass of MNs dried at 100 °C); S is the mass ratio of proline to silk fibroin; C is the silk fibroin concentration (g/mL) obtained by measuring the absorbance corresponding to the concentration-absorbance standard curve; and V is the buffer liquid volume of PBS (mL).

### 2.7. Morphology of MNs

The MNs before and after swelling were rapidly frozen in liquid nitrogen to maintain their expansion state. After vacuum drying, the needles had been cut off with a blade. The needle was sprayed with gold for 90 s, and the cross-section morphology was observed by Hitachi S-4800 scanning electron microscope (SEM) at 10 kV. The pore size was analyzed with Image J software.

Olympus laser confocal microscope (CLSM) was used to observe the drug distribution of the needle. The blue channel was selected, the excitation wavelength was set as 405 nm, and the acquisition wavelength was set as 435~480 nm.

### 2.8. In Vitro Release Properties

The MNs were inserted into the ventral skin of depilated SD rats, and the tip was fixed downward in the middle of the diffusion pool. Twelve milliliters of PBS solution was added to the diffusion tank, the water bath temperature was 32 °C, and rotate speed was 300 r/min. The drug release time was set as 8 h, sampling times were 16 times, and 1 mL sample was taken periodically, and at the same time, 1 mL of a fresh PBS buffer solution was added into the diffusion cell. Horiba FluorOMAX-4 fluorescence spectrometer was used to detect the release of melatonin. The excitation wavelength was 286 nm, the emission wavelength was 352 nm, the slit width was 5 nm, and the injection volume was 200 μL. The cumulative drug release ratio of transdermal drug release in vitro was calculated by the following formula:

(3) $$Q=\frac{C_{n}\;\times \;V+\sum_{i=1}^{n-1}C_{i}\;\times \;Vi}{M}\;\times \;100\%$$

where Q (%) is the cumulative drug release ratio of MN transdermal release; V is the volume of the receiving chamber (mL); Vi is the volume of each sample (mL); C_n_ and C_i_ are the drug concentration (μg/mL) in the receiving chamber; and M is the drug loading amount (μg).

The drug release rate of the MN in vitro was calculated according to the following formula:

(4) $$S=\frac{Q_{n}{\;\times \;V\;-\;Q}_{n-1}\times V}{T_{n}}$$

where S is the transdermal release rate of MN (μg/h); Q_n_ and Q_n−1_ were the cumulative drug release rates; V is the volume of the receiving chamber (mL); and Tn is the sampling interval (h).

### 2.9. In Vivo Animal Experiments

Modeling of insomnia rats: male SD rats were divided into standard groups and model groups. p-Chlorophenylalanine (PCPA) was prepared into 40 mg/mL PCPA suspension with weakly alkaline normal saline. The standard group was intraperitoneally injected with normal saline for 3 days, and the model group was intraperitoneally injected with PCPA suspension for 3 days. Compared with the standard group, the circadian rhythm of the rats in the model group disappeared, and the rats changed from interrupted sleep to restlessness during the day, indicating that the modeling was successful.

The insomnia rat model group was divided into blank group and drug administration group. Two administrative methods were used for comparison: intraperitoneal injection group (IP) and microneedle group (MN). Although intravenous administration usually results in the highest bioavailability of drugs, this route is generally impractical for rodents [37]. IP group was injected intraperitoneally with a syringe (70 μg), and the volume of the drug solution was 140 μL. In the MN group, the MN with 70 μg melatonin was inserted into the back of the depilated rats. Ten percent chloral hydrate solution was prepared with deionized water and injected intraperitoneally at a 3 mL/kg ratio. After anesthesia, the back hair of the rats was shaved with a shaving knife, the back skin was wiped with medical alcohol tablets, and the surface villi were carefully scraped with a shaving knife. Two hours later, when the rat was awake and back to normal, the microneedle was pressed on the rat’s back and fixed with medical tape. The experiment time was set as 6 h, and samples were taken at 0, 0.17, 0.33, 0.5, 0.83, 1.16, 1.5, 2, 3, 4, and 6 h, respectively. Twenty microliters of tail vein blood was collected from the tail vein of rats and centrifuged at 3000 rpm for 10 min. The separated supernatant was the serum. Plasma melatonin levels were determined by enzyme-linked immunosorbent assay (ELISA) using rat melatonin ELISA Kit (96T, Wuhan Moshak Biotechnology Co., Ltd., Wuhan, China). The absorbance was measured at 450 nm using a Synergy HT microplate reader, and a standard curve was drawn. Blood melatonin concentration was calculated from the standard curve.

All animals were kept in a pathogen-free environment and fed freely. The Ethics Committee approved the procedures for care and use of animals of the Soochow University Animal Center. All applicable institutional and governmental regulations concerning the ethical use of animals were followed.

### 2.10. Pharmacokinetic Analysis of Melatonin

The maximum plasma melatonin concentration (C_max_) and the time point of maximum plasma melatonin concentration (T_max_) were obtained from the relationship curve of plasma melatonin concentration with time. Relative bioavailability (RBA) was calculated using the following formula:

(5) $$\text{RBA}\left(\%\right)=\frac{{\text{AUC}}_{\text{MNs}}{\;\times \;\text{Dose}}_{\text{IP}}}{{\text{AUC}}_{\text{IP}}{\;\times \;\text{Dose}}_{\text{MNs}}}\times 100$$

where AUC_MNS_ is the area under the concentration–time curve of blood drug after MN administration; AUC_IP_ is the area under the concentration–time curve of blood drug after intraperitoneal injection of melatonin; Dose_MNS_ is the weight of the loaded drug in microneedle; and Dose_IP_ is the weight of the injected drug.

### 2.11. Statistical Analysis

The results were expressed as mean ± standard deviation. A difference of *p* < 0.05 was considered statistically significant.

## 3. Results

### 3.1. Structure of MT/SF@MNs

**Figure 3:**
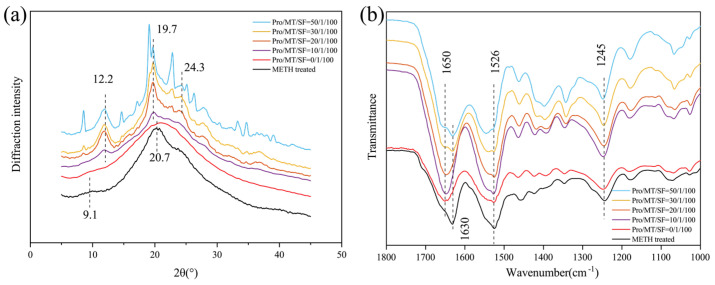
Structural analysis of MT/SF@MNs: (**a**) X-ray diffraction curve, (**b**) infrared absorption spectrum.

In the X-ray diffraction curve (Figure 3a), the crystal peaks of Silk I are 12.2°, 19.7°, and 24.7°, and those of Silk Ⅱ are 9.1° and 20.7° [38]. The 0/1/100 group of MNs showed an amorphous structure, and no crystallization peak appeared. This may be because melatonin is a water-insoluble drug [39]. After isopropyl alcohol assisted dissolution, it was mixed with silk fibroin solution in liquid form, effectively dispersing melatonin in silk fibroin solution. Studies have shown that isopropyl alcohol has little effect on the crystalline structure of silk fibroin solution [40]. The amount of isopropyl alcohol added in this paper was small, so silk fibroin remained in the solution state, sufficient for further MN preparation. Isopropyl alcohol was gradually volatilized during the drying process, and solid melatonin was evenly dispersed in the MN. Due to lack of water, although there was a specific interaction between melatonin and silk fibroin molecular chain, it was difficult to change the crystal structure of silk fibroin. At the same time, due to the low melatonin supplemental level, the silk fibroin MN eventually showed an amorphous structure.

After 90% methanol treatment, the SF@MN showed a very weak diffraction peak around 9.1° and a strong diffraction peak around 20.7°, indicating that SF gradually changed from random coil structure to Silk Ⅱ crystal structure. This is because the HLB value of small molecule is less than 10, which fails to provide a sufficient hydrophilic environment for SF and make SF molecules change to a more stable crystalline structure during hydrophobic action [41,42]. When proline was added to silk fibroin, crystallization peaks appeared near 12.2° and 19.7° when the mass ratio was 10/1/100. With the increase in proline ratio, the crystallization peaks of 12.2° and 19.7° became sharper, and a new crystallization peak appeared at 24.3°. The addition of proline changed the crystal structure of SF, and the random coil structure gradually changed to Silk Ⅰ crystal structure. Pro/MT/SF = 50/1/100, proline appeared self-crystallization, and the compatibility between proline and silk fibroin decreased.

In the infrared spectrogram (Figure 3b), the SF@MNs with only melatonin added had characteristic peaks near 1650 cm^−1^, 1526 cm^−1^, and 1245 cm^−1^, indicating that silk fibroin molecules were mainly random coil structures [43], which corresponded to the XRD results. This was also caused by using isopropyl alcohol to dissolve melatonin. The primary structural mechanism was as described in the XRD crystal structure above. After 90% methanol treatment, the typical β-sheet absorption peak appeared at 1630 cm^−1^, and the absorption peak was sharper near 1526 cm^−1^. This indicated that the addition of methanol rearranged hydrogen bonds in the random coil material, causing the transition of silk fibroin molecules from random coil to β-sheet [44]. With the increase of proline, the absorption peak at 1650 cm^−1^ gradually weakened, and a prominent peak appeared near 1630 cm^−1^. These results indicated that the addition of proline caused the secondary structure of SF@MN to transform from random coil or α-helix to β-sheet [45].

### 3.2. Mechanical Properties of MT/SF@MNs

**Figure 4:**
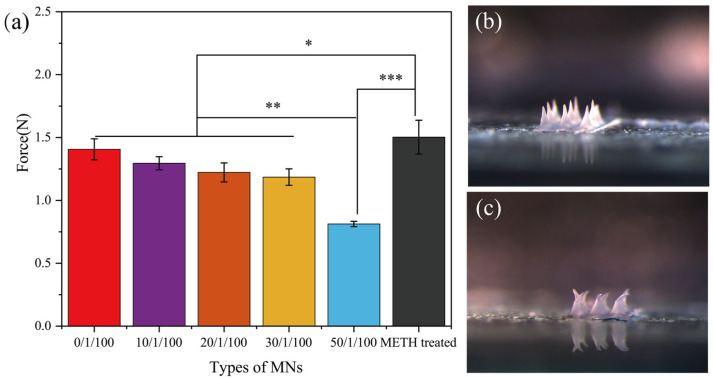
Compression performance test of MT/SF@MNs: (**a**) breaking strength tested by TMS-PRO texture instrument, (**b**) image of MNs image before compression, and (**c**) image of MNs after compression (* *p* < 0.05, ** *p* < 0.01, *** *p* ≤ 0.001).

Figure 4a shows the compressive breaking strength of MT/SF@MNs. The fracture strength of the MNs without proline modification and methanol treatment was high. With the increase in proline addition, the breaking strength of the needles gradually decreased, and the mechanical properties were slightly reduced. When Pro/MT/SF = 50/1/100, the mechanical properties of MNs decreased significantly. This was because proline content was too much, and more proline crystal was formed in the MN matrix, which can also be seen from the XRD curve. However, as a small amino acid, the crystalline aggregates of proline do not have strong mechanical properties, which are far inferior to those of silk fibroin. Figure 4b,c clearly showed the morphologic changes of the MNs before and after compression. After compression, the tip part of the needle was bent and fractured, which reflected the compressive fracture strength of the MN.

### 3.3. Penetration Performance of MT/SF@MNs

**Figure 5:**
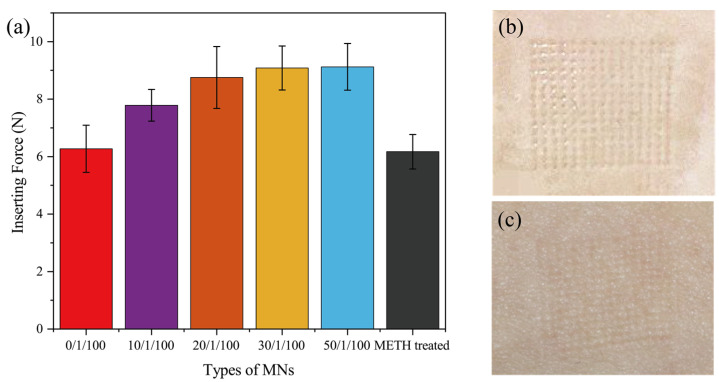
Penetration performance test of MT/SF@MNs: (**a**) penetration force of different MNs, (**b**) rabbit skin after penetration, and (**c**) rabbit skin 5 min after the removal of MNs.

The penetration force of MNs is shown in Figure 5a. MT/SF@MNs had good strength and penetrated easily into the skin. Pure SF@ MNs were treated with methanol to form the crystal structure of Silk Ⅱ. The mechanical properties of MNs were slightly improved, and they could penetrate the skin with less force. After proline modification of silk fibroin, mechanical properties of MNs decreased compared with pure SF@MNs, requiring more strength to penetrate the skin. With the increase in proline, the penetration force of MNs increased gradually. The penetration force of MNs was less than 10 N. The average single needle load was less than 0.05 N. Figure 5b shows that there were uniform pinholes on the skin surface of the rabbit after penetration, which was consistent with the number of needles. This indicates that the rate of penetration was 100%. After the MN was pulled out (Figure 5c), no redness or swelling appeared on the surface of rabbit skin, and the pinhole gradually disappeared 5 min later.

**Figure 6:**
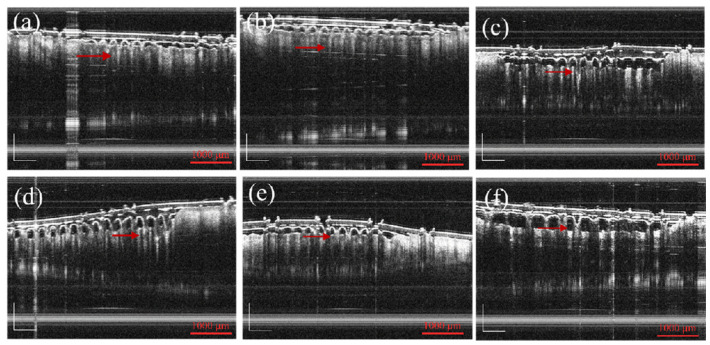
Optical coherence tomography images of MNs: (**a**–**f**) METH-treated Pro/MT/SF = 0/1/100, 10/1/100, 20/1/100, 30/1/100, and 50/1/100 (The red arrows indicate the MNs, scale bar is 1000 μm).

By observing the optical coherence tomography (OCT) images, it could be seen that the MNs evenly penetrated on the skin, and the penetration rate of MNs was 100%. This was consistent with the above skin penetration results. According to the OCT diagram in Figure 6, it was observed that with the increase in the Pro ratio, the skin depth of penetration entering the skin also decreased. Pro has a hygroscopic property, and the hardness of MN decreases due to this hygroscopic property. The effect of instantaneous puncture was not as good as that of the unmodified and methanol treated groups. When the Pro/MT/SF ratio was 50/1/100, there was a large gap between the chip and the skin during penetration. When the ratio was 10/1/100, 20/1/100, and 30/1/100, MNs had good adhesion. The MNs prepared in this experiment could penetrate the skin without producing a pricking sensation.

### 3.4. Swelling and Dissolution Loss of MT/SF@MNs

**Figure 7:**
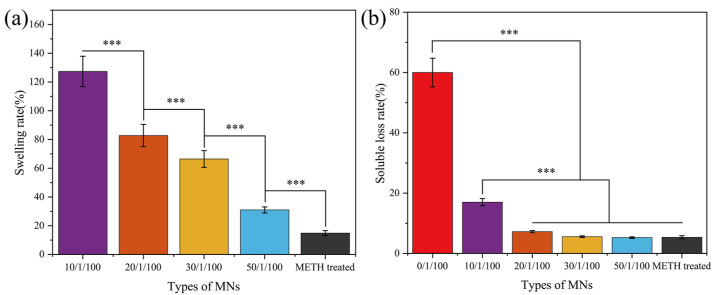
Swelling and dissolution loss of MT/SF@MNs’ (**a**) swelling rate and (**b**) soluble loss rate measured by ultraviolet spectrophotometer. (*** *p* ≤ 0.001).

As a small molecule additive, proline can adjust the swelling and dissolution performance of SF@MNs [34]. It is expected to achieve appropriate swelling and dissolution rates by adding different proline proportions. Figure 7 shows each group’s swelling and dissolution properties after the SF@MNs were shaken at 37 °C for 24 h. The 0/1/100 group had a dissolving rate of up to 60% (the swelling rate could not be tested), and the needle body dissolved and could not be used typically. After methanol treatment, the swelling rate and dissolution rate of MNs decreased considerably, the swelling rate was about 20%, and the soluble loss rate was only 5%. Combined with XRD and IR spectra, the SF@MNs treated with methanol formed a stable crystal structure of Silk II, making it difficult for water molecules to enter the interior. With the increase in proline addition, the microneedle’s swelling rate and dissolution rate also decreased correspondingly (Figure 7). When the ratio was 10/1/100, the swelling degree of MNs was higher, and the soluble loss rate was 17%. When the ratio was higher than 20/1/100, the MNs showed low dissolution. This was because the addition of small molecule proline made microneedles’ crystallization higher. When immersed in PBS solution, the MNs absorbed some water and swelled. A small crystallization zone inhibited its infinite swelling and did not dissolve.

MNs with ratios of 10/1/100, 20/1/100, and 30/1/100 were selected to build drug release platforms to achieve good penetration effect and swelling degree, reducing the dissolved substances in the body by considering the practical application of microneedles.

### 3.5. The Morphology of MT/SF@MNs

**Figure 8:**
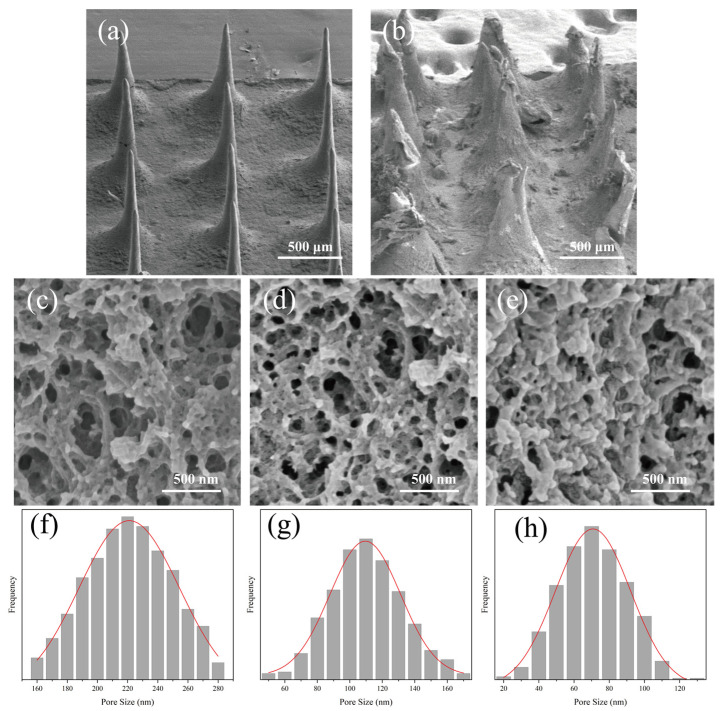
SEM images of the surface and section morphology of MT/SF@MNs: (**a**,**b**) surface images before and after microneedle swelling of Pro/MT/SF = 10/1/100, (**c**–**e**) cross-section images of Pro/MT/SF = 10/1/100, 20/1/100, 30/1/100 after microneedle swelling, and (**f**–**h**) pore size frequency distribution datagrams of Pro/MT/SF = 10/1/100, 20/1/100, 30/1/100 after microneedle swelling.

To compare the morphologic changes of the MNs before and after swelling, electron microscope images of the surface and cross-section of the needle body were taken (Figure 8). The aperture of each microneedle is summarized in Table 1. The surface morphology of MNs before and after swelling is shown in Figure 8a,b. After swelling, there were swelling traces of the MNs, the structure was looser than before soaking, and there was apparent layered structure accumulation on the surface. Further observation of the internal structure of the micropuncture can be seen in Figure 8c–e. There were pores in the internal network, and with the increase om the proline ratio, the internal structure became denser, and the aperture range also decreased. The pore size of the MNs showed a linear relationship with the amount of proline. The pores formed by the swelling of SF@MNs could provide channels for drug molecules, and drugs showed different release rates with other swelling properties. Based on this principle, we can build a microneedle controlled release drug delivery system to control the drug release, reduce the risk, and improve the safety for using.

### 3.6. In Vitro Release of MT/SF@MNs

To discuss the melatonin release, MNs were inserted into the skin of rats to study the cumulative drug release rate in PBS solution.

**Figure 9:**
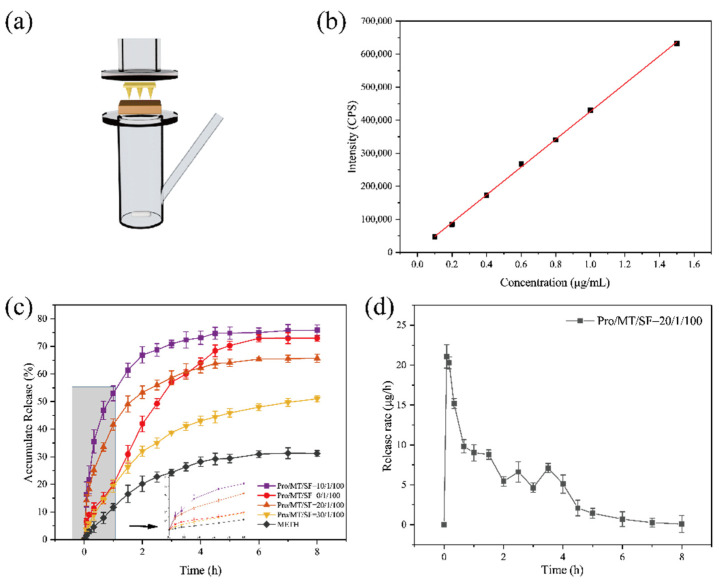
In vitro drug release of MT/SF@MNs: (**a**) in vitro drug-release setup, (**b**) standard curve of melatonin fluorescence detection, (**c**) cumulative release curve of MT/SF@MNs, (**d**) release rate curve of Pro/MT/SF = 20/1/100 MNs, and (**c**,**d**) were tested by Horiba FluorOMAX-4 fluorescence spectrometer.

According to Figure 9c, the drug release of all microneedles reached equilibrium after 8 h. The cumulative release rate of all microneedles experienced a change process of first fast and then slow. As the structure of the unmodified MN was the mainly random coil, the MN wrapped with melatonin would be dissolved in water when entering the skin, and the drug in the tip would be released quickly. The drug release balance was reached within 5 h, with a cumulative drug release rate of 72.96%. Dissolved microneedles can be used for rapid drug release, but at the same time, the components making up the microneedles also enter the skin. Even though SF has good biocompatibility, the effects of long-term accumulation in the skin are unknown.

After 90% methanol treatment, SF changed from random coil to Silk Ⅱ, with low swelling and dissolution rates. At this time, the drug release was slow, and the drug release amount was small, which could not be applied effectively. The drug release equilibrium was reached in 5 h, and the cumulative drug release rate was 31.21%.

When Pro/MT/SF = 10/1/100, the drug release amount and release rate were the largest, and compared with other MNs, the drug amount was utilized to the maximum, and the release balance was reached at 4 h, with the cumulative drug release rate of 75.88%. In Pro/MT/SF = 20/1/100, the drug reached release equilibrium at 6 h, and the cumulative release rate was 65.71%. When the proline content was relatively low, the drug release curve of modified MNs was more inclined than that of pure silk fibroin; that is, the release rate of modified MNs was faster than that of soluble MNs. MT is lipophilic, and the dissolution rate in SF solution is meagre [46]. In this paper, a small amount of isopropyl alcohol solution was added to assist the dissolution of MT so that MT could be evenly distributed in the SF solution. When the proline content was low during drying, there was less cross-linking with silk fibroin, resulting in a small amount of melatonin accumulating on the microneedle’s surface with the volatilization isopropyl alcohol. After contact with the liquid, the melatonin on the surface of the microneedle will be released quickly. In contrast, the melatonin in the needle body will be released slowly with the swelling of the microneedle. When Pro/MT/SF = 30/1/100, there was more cross-linking inside the microneedle, and the release rate of melatonin was slow. The release balance was reached at 8 h, and the cumulative release rate was 51.01%, indicating a low drug utilization rate.

Insomnia treatment requires long-term maintenance of melatonin levels to mimic its physiological release. Therefore, a sustained release formulation of exogenous melatonin needs to cover the entire nighttime cycle to improve sleep disorders. When the ratio was 10/1/100, MN had a higher dissolving rate. When the ratio was 30/1/100, the utilization rate of MT was low. Therefore, Pro/MT/SF = 20/1/100 MNs were selected for animal experiments.

The drug release rate of the MN with Pro/MT/SF = 20/1/100 is shown in Figure 9d. It is clear from the figure that the drug release rate was the fastest within the first 10 min, indicating that melatonin could quickly reach the body through the epidermis. In the first 4 h, the drug continued to release at a high rate, and in the later period, the release rate gradually decreased. This suggests that MT/SF@MNs could control the release rate of drugs, allowing the sudden release of drugs in the early stage and stable release rate in the later stage, covering the entire sleep cycle.

### 3.7. Distribution of Melatonin in the Needle

**Figure 10:**
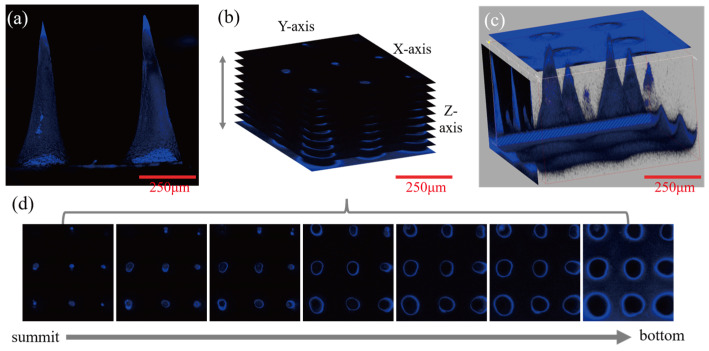
Confocal laser microscopy photos of the 20/1/100 microneedle: (**a**) CLSM images of single-layer scanning when the body of melatonin microneedle was parallel to the objective lens; (**b**) z-axis continuous rotation (x: 306.5°, y: 301.3°, z: 57.6°) vertical accumulation; (**c**) 3D synthetic images of melatonin microneedles under CLSM; (**d**) CLSM images of the xy-plane in the z-axis series when the tip of the melatonin microneedle is facing the objective lens. (Scale bar is 250 μm).

Laser confocal microscopy (CLSM) was used to scan the 20/1/100 microneedle group from the tip to the base to observe the distribution of melatonin in the needle (Figure 10). It could be observed from the fluorescence imaging of the MT that melatonin was evenly distributed on the surface, as shown in Figure 10a. This finding was also supported in CLSM analysis (Figure 10b,d). The distribution of MT was uniform in the xy-plane (horizontal) and z-axis continuous (vertical) fluorescence images. Because of the layer scanning with different focal lengths, the fluorescence signal of melatonin could be evenly distributed and enriched on the surface of the microneedle. It was less spread in the interior of the microneedle in the xy-plane microscope photos of the z-series layered arrangement. The microscope photos were rotated and stacked together as shown in Figure 10b, and the 3D synthesis of melatonin microneedles under CLSM could be intuitively observed, as shown in Figure 10c.

### 3.8. In Vivo Experiment of MT/SF@MNs

**Figure 11:**
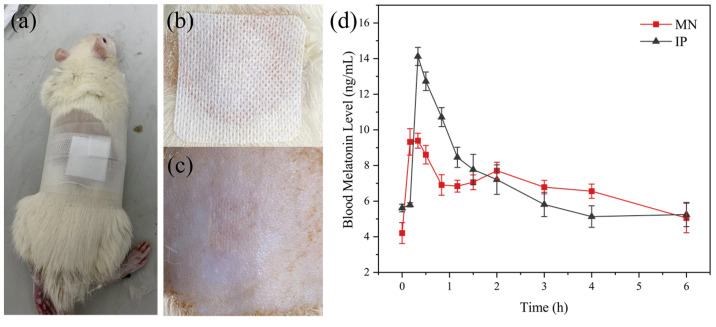
Treatment of insomnia rats with melatonin microneedles: (**a**,**b**) pictures of a rat treated with MT/SF@MNs, (**c**) pictures of rat skin after removal of MN, and (**d**) curves of blood melatonin concentration over time.

Compared with the regular group, the rats in the modeling group showed restlessness, and the circadian sleep rhythm disappeared. For studying the treatment of MT/SF@MNs to insomnia, the model group was treated with melatonin. The process of MN treatment is shown in Figure 11a,b. The rats quickly entered the sleep state and remained in the sleep state during the treatment. At the end of drug administration, the MN patch was removed, no redness and swelling were observed on the skin attached to the MN, and no adverse reactions were observed in rats at the later stage (Figure 11c). The rats in the MN group and IP group entered the sleep state quickly.

The change curve of blood melatonin concentration over time in each group is presented in Figure 11d. In the two experimental groups, melatonin was rapidly absorbed soon after administration, and the blood melatonin content in vivo quickly increased, which was consistent with the “sudden release” phenomenon of previous drugs in vitro (Figure 9d). With the extension of time, the curve fluctuation of the MN group was gentler than that of the IP group, and the blood melatonin concentration was not low until 4 h, which was consistent with the release rate of melatonin in vitro release.

According to Figure 11d, it could be found that melatonin delivered by MN can quickly reach the maximum blood concentration while maintaining a high level in the body for a long time. The peak time was 0.31 ± 0.07 h in the MN group and 0.41 ± 0.09 h in the IP group. The mean maximum blood concentration C_max_ was 10.08 ± 1.26 ng/mL in the MN group and 15.51 ± 1.88 ng/mL in the IP group. Despite the same melatonin dose in both preparations, C_max_ (10.08 ng/mL) in the MN group was lower than in the IP group. This suggests that the controlled-release component of the MN leads to a slower release of MT, thus avoiding the significant surge effect that might occur with the fast-release tablet and revealing the potential of the microneedle delivery system for continuous delivery of melatonin.

The pharmacokinetic parameters of the two groups with different administration methods are shown in Table 2. According to Table 2 and Formula (4), the relative bioavailability of the MN group to the IP group was about 68.05%. The area under the concentration–time curve (AUC) of the MN group was lower than that of the IP group, both to the last sampling time (AUC_0-tlast_) and extrapolated to infinity (AUC_0__–∞_), which was consistent with pharmacokinetics. Long-term use of melatonin at superphysiological doses may increase the potential risk of adverse melatonin reactions. Blood melatonin concentrations in the MN group did not jump to such high levels as to pose this potential risk.

## 4. Discussion

Insomnia has become a significant problem that cannot be ignored in today’s society. In this study, we prepared the silk fibroin microneedle patch loaded with melatonin to treat insomnia for the first time. For controlling melatonin’s release rate, proline was selected to modify silk fibroin to transform from random coil structure into Silk Ⅰ crystal structure. When the dosage of proline was 1050 wt% (relative to the weight of silk fibroin), the compressive strength of 3 × 3 array microneedles was 0.8–1.4 N, and the single puncture force was less than 0.05 N, which could meet the requirements of penetrating the skin surface without causing pain. The swelling capacity of the modified microneedle was significantly increased (30–127%), and the dissolution rate was less than 20%. Previous work has verified that the internal pore size of microneedles with different swelling degrees varies significantly after expansion, thus affecting the release rate and time of drugs [34].

**Figure 12:**
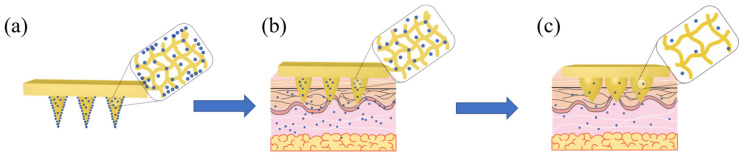
Release mechanism diagram of MT/SF@MNs: (**a**) schematic diagram of original microneedles, (**b**) schematic diagram of the early stage of microneedles, and (**c**) schematic diagram of the late stage of microneedles.

The drug-release ability of SF@MNs with a different swelling degree was evaluated in vitro. When the proline content was low, a small amount of MT was assembled on the surface of the needle, and the drug release rate was faster in the early stage, even higher than that of the pure SF@MNs group. When the proline content increased, the crystallinity of the microneedle increased, the dissolution rate decreased, and the internal pore size decreased, which limited the rapid release of the drug. Therefore, according to this feature, we drew the internal structure changes and drug release mechanism of proline-modified MN, as shown in Figure 12. Before the drying of the microneedle, melatonin was evenly distributed in the SF solution. After drying, part of the drugs accumulated on the surface of the microneedle with the volatilization of isopropyl alcohol (Figure 10d). The rest was wrapped in the interior of the needle by silk fibroin molecules (Figure 12a). As soon as the skin was micropunctured with the designed microneedle, the drug on the surface was rapidly released into the body. The microneedle was absorbed the body fluid and became slightly swollen. The internal pore size was increased, and the drug molecules inside began to move and enter the body along the concentration difference (Figure 12b). The proline modified microneedles, with Silk Ⅰ structure as the main structure (Figure 3a), could further swell. The internal pore size continued to increase, resulting in the continuous release of drugs. At the same time, due to the existence of a certain amount of α-helix in its interior, the solid intermolecular interaction limits the infinite swelling of the microneedle (Figure 7a), preventing it from causing a large amount of dissolution (Figure 7b). The drug can be released at a certain speed in a later period (Figure 12c).

Pro/MT/SF = 20/1/100 microneedles were selected for in vivo experiments in animals based on carefully considering mechanical properties, dissolution loss, and in vitro drug release rate. When the MN was inserted into the skin of rats, the melatonin that accumulated on the surface of the needle body was rapidly dissolved in body fluids, and the release rate was maintained at a high rate in the early stage, making the blood drug concentration of rats rise quickly. In the later stage, MN absorbed body fluid and swelled. Meanwhile, an internal crystallization region limited the infinite swelling of the MN, and the inner drugs decreased in concentration. The blood drug concentration in rats increased, and the concentration gradient decreased, decreasing the drug release rate. Since the half-life of melatonin in vivo is about 30 min, the amount of drug released by MN is relatively balanced with the amount of drug reduced in rats, so that the blood concentration of melatonin in rats can be maintained at 7–8 ng/mL, which can help to achieve good sleep regulation.

In this paper, the combination of microneedle therapy and melatonin administration for the first time showed excellent results in the treatment of insomnia. Among the existing melatonin dosage forms, oral dosage forms had low bioavailability (about 15%) and liver first-pass effect [47]. Some researchers used nasal mucosal melatonin administration and achieved the same bioavailability as intravenous injection [18,48]. However, this method could not reach a sustained release effect, although the drug acted quickly and could only maintain continuous administration for 2 h. The nasal mucosal drug delivery system had local tolerance, systemic and central nervous side effects, and pulmonary effects [49]. Some researchers also used a non-microneedle skin patch, which achieved good sleep regulation and neuroprotective measures. However, this non-microneedle skin patch had a slow effective rate, with the maximum diffusion rate reaching about 2–4 h [50], which is slower than the microneedle patch prepared in this paper. Compared to the existing melatonin administration schemes, the microneedle administration has achieved a good combination of bioavailability, drug onset time, and sustained-release effect. We consider that this system can successfully be applied to treat insomnia after completing pre-clinical and clinical investigations.

## 5. Conclusions

A melatonin-loaded silk fibroin microneedle patch was prepared for the treatment of insomnia. The microneedle had a uniform shape and high enough strength to penetrate the cuticle of the skin easily. Optical coherence tomography observation showed that the microneedles penetrated 100% of the human skin without breaking. There was no residual material in the body after the microneedles were pulled out. The melatonin-loaded silk fibroin microneedles (MT/SF@MNs) could rapidly release melatonin early and maintain a steady release rate for 4–6 h in the later stage. The results of microneedle therapy in rats with insomnia showed that the microneedle (MN) group could quickly reach the effective blood concentration of melatonin, make the rats enter the sleep state quickly, and prolong the adequate time of the blood concentration to 6 h. The silk fibroin microneedle delivery system accelerates the time for melatonin to enter the blood. This improves the bioavailability of melatonin and reduces the risk of excessive drug concentration. After conducting the relevant pre-clinical and clinical investigation, it is expected to have a perfect space for developing new strategies for insomnia treatment.

## Figures and Tables

**Table 1:** Pore size of Pro/MT/SF in different proportions.

Type of MNs	Pro/MT/SF = 10/1/100	Pro/MT/SF = 20/1/100	Pro/MT/SF = 30/1/100
Pore size (nm)	223.6 ± 29.3	117.3 ± 24.1	74.8 ± 18.6

**Table 2:** Pharmacokinetic parameters of rats in the two groups after drug treatment.

	IP	MN
T_max_ (h)	0.41 ± 0.09	0.31 ± 0.07
C_max_ (ng/mL)	15.51 ± 1.88	10.08 ± 1.26
AUC_0-tlas_*_t_* (h·ng/mL)	61.07 ± 4.37	41.56 ± 1.34
AUC_0__–__∞_ (h·ng/mL)	65.32 ± 9.44	50.58 ± 6.65
RBA (%)	100	68.05 ± 3.47

## Data Availability

Not applicable.

## Abbreviations

SF	silk fibroin
MN	melatonin
Pro	proline
MT/SF@MNs	melatonin-loaded silk fibroin microneedles
OCT	optical coherence tomography
SD	Sprague dawley
IP	intraperitoneal injection group
C_max_	maximum blood concentration
AUC	area under the concentration–time curve
T_max_	peak time
